# Cord blood versus age 5 mononuclear cell proliferation on IgE and asthma

**DOI:** 10.1186/1476-7961-8-11

**Published:** 2010-08-04

**Authors:** Carolyn Chang, Kevin Gauvey-Kern, Alina Johnson, Elizabeth A Kelvin, Ginger L Chew, Frederica Perera, Rachel L Miller

**Affiliations:** 1 Division of Pulmonary, Allergy and Critical Care Medicine, Columbia University College of Physicians & Surgeons, New York, NY, USA; 2 Department of Environmental Health Sciences, Mailman School of Public Health, Columbia University, New York, NY, USA; 3 Department of Pediatrics, Columbia University College of Physicians & Surgeons, New York, NY, USA

## Abstract

**Background:**

Fetal immune responses following exposure of mothers to allergens during pregnancy may influence the subsequent risk of childhood asthma. However, the association of allergen-induced cord blood mononuclear cell (CBMC) proliferation and cytokine production with later allergic immune responses and asthma has been controversial. Our objective was to compare indoor allergen-induced CBMC with age 5 peripheral blood mononuclear cell (PBMC) proliferation and determine which may be associated with age 5 allergic immune responses and asthma in an inner city cohort.

**Methods:**

As part of an ongoing cohort study of the Columbia Center for Children's Environmental Health (CCCEH), CBMCs and age 5 PBMCs were cultured with cockroach, mouse, and dust mite protein extracts. CBMC proliferation and cytokine (IL-5 and IFN-γ) responses, and age 5 PBMC proliferation responses, were compared to anti-cockroach, anti-mouse, and anti-dust mite IgE levels, wheeze, cough, eczema and asthma.

**Results:**

Correlations between CBMC and age 5 PBMC proliferation in response to cockroach, mouse, and dust mite antigens were nonsignificant. Cockroach-, mouse-, and dust mite-induced CBMC proliferation and cytokine responses were not associated with allergen-specific IgE at ages 2, 3, and 5, or with asthma and eczema at age 5. However, after adjusting for potential confounders, age 5 cockroach-induced PBMC proliferation was associated with anti-cockroach IgE, total IgE, and asthma (p < 0.05).

**Conclusion:**

In contrast to allergen-induced CBMC proliferation, age 5 cockroach-induced PBMC proliferation was associated with age 5 specific and total IgE, and asthma, in an inner-city cohort where cockroach allergens are prevalent and exposure can be high.

## Background

There has been longstanding controversy in the literature regarding whether biomarkers measured in cord blood may help predict subsequent childhood asthma or atopy [1-9]. Prospective birth cohorts studies have demonstrated that cord blood IgE is a better predictor of skin prick test (SPT) positivity to aeroallergens (dust mite, grass, cat and dog) than family history when assessed up to age 5 years [2,6,7]. A similar positive association with early asthma at age 5 years has been more difficult to demonstrate [2,7]. However, others have shown a positive association between elevated cord blood IgE with risk of later asthma at age 10 years [9], and allergic rhinoconjunctivitis at age 20 years [8].

The findings continue to be mixed when comparing aeroallergen-induced cord blood mononuclear cell (CBMC) proliferation with the subsequent risk for developing asthma, eczema, and allergic rhinitis [10-12]. At birth, infants who developed allergic disease by age 1 year had significantly more positive CBMC responses to dust mite and food allergen proteins than newborns who did not develop allergy [13]. Notably, one birth cohort that followed children as long as 6 years demonstrated no significant difference in aeroallergen-induced (dust mite, grass, mold, cat) CBMC proliferation among cord blood samples of children who subsequently developed atopic disease by 6 years of age compared with samples from children who did not [14]. It has been argued that allergen-induced CBMC proliferation may represent a default immune response by recent thymic emigrants as opposed to a more mature T cell memory response [15]. However, other approaches, such as those using MHC tetramer staining, have demonstrated antigen specific intrauterine T cell immune response following environmental exposures that display features of immunologic effector memory [16].

Only a few studies have reported on mitogen or antigen-induced CBMC T helper (Th) cytokine production, and compared their levels with the likelihood of later atopy. For example, increased phytohemagglutinin (PHA)-induced interleukin (IL)-5 and IL-13 was associated with increased total IgE during the first year of life [17]. Dust mite-induced CBMC production of IL-13 was associated with SPT positivity in response to dust mite antigens at age 6 [10]. However, associations between dust mite-induced CBMC production of IL-6 and IL-10 and subsequent atopic disease (i.e. asthma, eczema) or SPTs at age 6 years were absent [10,18].

Despite this body of work, studies to date have not yet compared prospectively the association between antigen-induced lymphoproliferative responses in cord blood with repeat measures in later childhood, and assessed the relative strengths of their associations with childhood asthma or eczema. In addition, the roles of early immune responses following *ex vivo *stimulation with cockroach and mouse proteins, antigens associated with inner city asthma [19,20], have not been fully elucidated. Our objective, using a longitudinal birth cohort designed to examine risk factors for the development of asthma in an inner city population, was to compare cockroach, mouse and dust mite antigen-induced lymphoproliferative response in cord blood with age 5 antigen-specific lymphoproliferative response among the same children, and determine whether either were associated with a greater likelihood of age 5 atopy. We hypothesized that indoor allergen-specific cord blood proliferation and Th2 cytokine production would be associated with subsequent childhood (ages 2, 3, and 5) IgE, asthma, and eczema. We report that, in contrast to allergen-induced CBMC proliferation, age 5 cockroach-induced PBMC proliferation was associated with age 5 specific and total IgE, and asthma, in an inner-city cohort where cockroach allergens are prevalent and exposure can be high.

## Methods

### Study subjects

As part of an ongoing longitudinal birth cohort study conducted under the auspices of the Columbia Center for Children's Environmental Health (CCCEH), women ages 18 to 35, living in Northern Manhattan and the South Bronx, were enrolled during pregnancy (n = 725) from clinics affiliated with New York Presbyterian Hospital (Columbia campus) or Harlem Hospital as described [19,21,22]. Exclusion criteria for pregnant women included smoking, illicit drug use, diabetes, hypertension, HIV infection, and residence in New York City for less than one year.

From this cohort of fully enrolled mothers, a sample based on the number of children from whom a blood sample was obtained (i.e. any time point from cord blood through age 5 years) was selected for inclusion (n = 609). For longitudinal analysis, a subset (n = 359) inclusive of all children for whom cord blood was collected and data were available for prospective analysis at age 2, 3 and 5 year was studied. For cross-sectional analysis, another overlapping subset (n = 352) inclusive of all children for whom age 5 blood was collected was assessed for concurrent (age 5) outcomes symptoms.

Written informed consent was obtained from all study participants and Columbia University's Institutional Review Board approved the study.

### Questionnaires

Detailed questionnaires were administered to women prenatally, every 3 months until the child was age 2, and every 6 months thereafter until age 5 [19,22]. Questionnaires assessed demographics, maternal asthma, environmental tobacco smoke (ETS) exposure, and report of wheeze, cough and physician diagnosis of asthma and/or eczema. Furthermore, at age 5, parental report of eczema was determined using the validated International Study of Asthma and Allergy in Childhood (ISAAC) eczema questionnaire [23-25], and parental report of asthma was determined using the locally validated Brief Respiratory Questionnaire (BRQ) [26].

### Home allergen measurements

Dust samples were vacuumed separately from kitchens and mothers' beds prenatally and were analyzed for mouse urinary protein (MUP), dust mite (Der f 1), and cockroach (Bla g 2) allergens by enzyme-linked immunosorbent assay (ELISA) as described [27-30].

### Blood collection, mononuclear proliferation, cytokine assays, and IgE

Cord bloods were collected at delivery and maternal blood within 1 day postpartum [19,21,22,31]. Peripheral blood samples at 2, 3 and 5 years of age were collected. Briefly, fresh mononuclear cells were isolated by density centrifugation and plated in triplicate for mononuclear proliferation and in duplicate for cytokine assays. Antigen-induced mononuclear cell proliferation and cytokine production were measured in cord blood and in peripheral blood at age 5 years.

Mononuclear cells (3 × 10^5 ^cells/well) were cultured in microtiter plates for 5 days with *Blatella germanica *(German cockroach; 10 μg/ml; Greer Laboratories, Lenoir, NC), *Dermatophagoides farinae *(dust mite; 10 μg/ml; Greer Laboratories, Lenoir, NC), *Mus musculus *(mouse protein extract; 10 μg/ml; Greer Laboratories, Lenoir, NC), or no antigen [21]. Increased proliferation in response to German cockroach, dust mite, and mouse protein extract antigens were detected by tritiated thymidine incorporation. Increased mononuclear cell proliferation were defined as (1) a stimulation index [SI] (averaged counts per minute [cpm] in the presence of antigen divided by averaged cpm without antigen) greater than 2, and (2) antigen-induced cpm greater than 1,000 above background [21]. Separate cell aliquots for cytokine analysis were cultured under identical conditions, and supernatants were collected at day 5 and analyzed in duplicates for IL-5 and IFN-γ via ELISA kits (Immunotech, Marseille, France) [21].

Anti-cockroach, anti-mouse, and anti-dust mite IgE levels were measured in sera initially by using the Fluorescence Allergosorbent Test (FAST) (Bio Whittaker, Walkersville, MD) until August 2002. Subsequently, all samples were measured by ImmunoCAP (Phadia, Uppsala, Sweden). Total IgE levels were measured initially by immunoradiometric assay (IRMA) (Total IgE IRMA; Diagnostics Products Corp, Los Angeles, CA), and subsequently (after August 2002) by ImmunoCAP (Phadia, Uppsala, Sweden). All samples were measured in duplicate and during the transition of one validated method to another, a subset was analyzed using both methods to ensure correlation of results, as previously described [19]. Antigen-specific IgE levels of 0.35 IU/ml or greater (class I) were considered positive.

### Statistical analyses

Data were analyzed with SPSS version 16.0 (SPSS, Inc, Chicago, Ill). Dust allergens levels were analyzed as natural logarithm-transformed continuous values and in tertiles. Mononuclear cell proliferation results were analyzed as continuous (antigen-induced cpm divided by background cpm) or dichotomous (positive versus negative SI) variables. Cytokine responses to cockroach, mouse, and dust mite antigens were measured as cytokine ratios: (measured response to antigen)/(measured response to background condition) and analyzed as continuous variables. Allergen-specific IgE levels were analyzed as dichotomous variables (≥0.35 IU/ml, <0.35 IU/ml). Total IgE and sum of allergen-specific IgE (sum of anti-cockroach, anti-mouse, and anti-dust mite IgE) were analyzed as continuous variables. The later approach was intended to study a derived indicator of allergic sensitization to indoor allergens, important to inner city asthma [19], with the benefit of greater statistical power. All values below limit of detection (LOD) were recoded as half LOD. Symptoms and diagnoses assessed by questionnaires were analyzed as dichotomous variables (yes or no). All continuous variables were natural log transformed.

Fisher's Exact Test and nonparametric tests, including Mann-Whitney U (MWU) Test, Kruskal-Wallis Test, and Spearman's rho correlation, were used to ensure data results were not distorted by failure to fulfill parametric distribution requirements. Unadjusted logistic and linear regression analyses were performed to assess whether allergen-specific mononuclear cell proliferation were significant predictors of atopy and asthma, eczema, allergen-specific IgE levels, and total IgE levels. Adjusted multivariate logistic and linear regression models were examined to adjust for (1) child's sex, (2) ethnicity, (3) any ETS exposure at home at age of interest, (4) maternal history of asthma, and (5) prenatal allergen levels in bed. Interaction terms also were examined between each independent variables of interest and each of the five covariates described above in order to differentiate between confounders and effect modifiers. Statistical significance was defined as a two-tailed p < 0.05.

## Results

### Study population, age-related indoor antigen-induced proliferation

Children were predominantly Dominican (63.8%) with lower socioeconomic status and frequent use of public assistance. Twenty one percent of the mothers reported asthma, whereas asthma diagnosis in the child ranged from approximately 15% to 17.7% at ages 2, 3 respectively, and occurred among 29.6% by age 5 years (Tables 1, 2).

**Table 1:** Characteristics of Study Children

	%	n/Total
Sex		
Female	52.0	316/608
Ethnicity		
Dominican	63.8	388/608
African American	36.2	220/608
Maternal History of Asthma	21.4	130/608
Prenatal ETS Exposure*	49.7	237/477
Postnatal ETS Exposure		
Age 2	43.5	202/464
Age 3	49.3	225/456
Age 5	59.2	239/404
Maternal Highest Degree		
High School Diploma or More	64.9	389/599
Currently on Medicaid	90.3	548/607
Currently in Public Assistance	42.3	255/603

Values shown are validated percents to account for missing variables.

ETS, environmental tobacco smoke

*In the absence of maternal smoking

**Table 2:** Childhood Symptoms at Ages 2, 3, and 5 Years

	Frequency (%)	n/Total
**Age 2**		
Asthma^1^	15.2	41/269
Wheeze^1^	15.3	41/268
Cough^1^	50.2	135/269
Eczema^1^	21.6	57/264
**Age 3**		
Asthma^1^	17.7	48/274
Wheeze^1^	11.8	32/272
Cough^1^	47.1	128/272
Eczema^1^	25.1	68/271
**Age 5**		
Physician Diagnosed Asthma ^2^	29.6	66/223
Wheeze/Whistling/Cough/Other ^2^	31.8	71/223
Cough^1^	49.2	119/242
Itchy Rash Last 12 months ^3^	65.9	29/44
Ever Had Eczema ^3^	27.9	62/222

Values shown are validated percents to account for missing variables.

Sample inclusive of children in whom we have cord blood biological data (n = 359).

Parental report of symptoms assessed by: ^1^Study Questionnaire, ^2^Brief Respiratory

Questionnaire, and ^3^ISAAC Eczema Module.

ISAAC, International Study of Asthma and Allergy in Childhood

Significant correlations between CBMC and age 5 PBMC proliferation in response to all three antigens examined were absent even after stratifying by antigen-specific maternal blood proliferation positivity (Table 3). In addition, maternal and cord blood mononuclear cell proliferation continued to differ with each other in response to cockroach as reported in 2001 [21] and now when reanalyzed in 2010 (2001: n = 133, p < 0.05 by Fisher's Exact Test; 2010: n = 277, p < 0.05 by Fisher's Exact Test). However, statistically differences between maternal and cord blood in the response to mouse antigen that were absent in 2001 were now detected in the larger data set (2001: n = 132, p > 0.05 by Fisher's Exact Test; 2010: n = 252, p < 0.05 by Fisher's Exact Test). In comparison, statistically significant maternal versus cord blood differences follow exposure to dust mite antigens detected in 2001 were not apparent in 2010 with the larger data set (2001: n = 131, p < 0.05 by Fisher's Exact Test; 2010: n = 259, p > 0.05 by Fisher's Exact Test) [21]. Furthermore, antigen-specific maternal blood mononuclear cell proliferation was not correlated with antigen-specific age 5 mononuclear cell proliferation (cockroach antigen n = 87, p = 0.82; mouse antigen n = 75, p = 0.33; dust mite antigen n = 80, p = 0.07l; by Fisher's Exact Test), suggesting that maternal antigen-induced peripheral blood mononuclear cell proliferation does not predict the development of specific antigen-induced T cell proliferative responses in their children through age 5 years.

**Table 3:** Correlation of Cord Blood and Age 5 Blood Antigen Specific Proliferation

		**Cord Blood**		
		**N**	**R**	*p-value
		
**Age 5 Blood**	**Cockroach**	97	-0.033	0.750
	**Mouse**	86	-0.074	0.5
	**Dust Mite**	88	-0.171	0.111

Analyses by Spearman's rho correlations, p-value two-tailed

N, number of observations

R, correlation coefficient

*Results did not differ after stratifying by antigen-specific maternal blood proliferation positivity.

### Cord blood proliferation, cytokine production, IgE and respiratory symptoms

**Figure 1:**
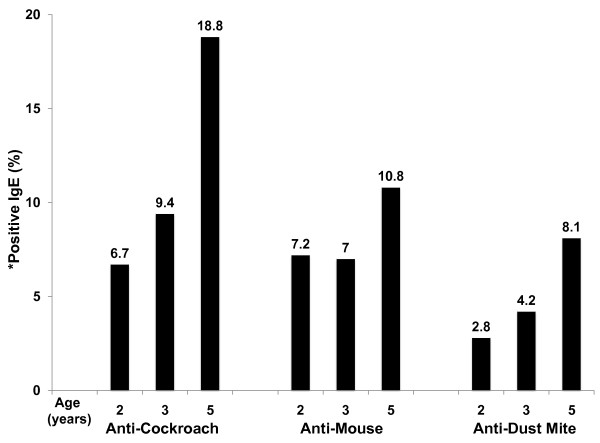
**Frequency (%) of Antigen-Specific IgE Positivity at Ages 2, 3, and 5**. *Anti-cockroach, anti-mouse, anti-dust mite IgE positivity is defined as ≥ 0.35 IU/ml.

By age 5 years, 18.8%, 10.8%, and 8.1% of children developed positive anti-cockroach, anti-mouse, and anti-dust mite IgE, respectively (Figure 1). However, cockroach-, mouse-, and dust mite-induced CBMC proliferation and IL-5 and IFN-γ cytokine production were not associated with antigen-specific or total IgE levels at ages 2, 3, or 5 (data not shown), suggesting that antigen-induced T cell allergic immune responses in CBMC are not associated with a greater likelihood of developing allergen-specific IgE responses in early childhood. To assess prospectively whether antigen-induced T cell proliferative responses were associated with asthma and eczema symptoms through age 5 years, CBMC proliferation was compared to the frequency of parental report of asthma, cough without a cold, wheeze and eczema. Antigen-specific CBMC proliferation was not associated with maternal report of child asthma, cough, wheeze or eczema at ages 2, 3, and 5 (p > 0.05).

### Age 5 proliferation, IgE and respiratory symptoms

To ascertain whether concurrent antigen-induced PBMC proliferation is associated with IgE outcomes at age 5 years, cross-sectional comparisons between age 5 year PBMC proliferation and total and allergen-specific IgE levels were conducted. In univariate analysis, cockroach antigen-induced PBMC proliferation was not associated with anti-cockroach IgE production (n = 113, OR 1.75, 95% CI 0.69, 4.42) (Table 4). However, after adjusting for potential confounders (sex, ethnicity, current ETS exposure, maternal asthma, prenatal cockroach allergen levels in bed), children with positive cockroach-induced PBMC proliferation had almost 3.4 times increased odds of positive concurrent anti-cockroach IgE (n = 78, OR 3.39, 95% CI 1.05, 10.92) compared to children without positive cockroach-induced PBMC proliferation (Table 4). In addition, significant interactions were detectable between age 5 cockroach-induced PBMC proliferation with ethnicity (p = 0.04) and sex (p = 0.01) on age 5 anti-cockroach IgE. In stratified adjusted models, the associations between cockroach-induced PBMC proliferation and anti-cockroach IgE at age 5 years was detected amongst African Americans (β = 1.86, p = 0.00) and girls (β = 1.05, p = 0.00).

**Table 4:** Age 5 Cockroach Antigen-Induced PBMC Proliferation and IgE at Age 5 years

	Outcomes			
	
	**Anti-cockroach IgE **≥ **0.35 IU/ml**		Total IgE (IU/ml)	
**Predictors**	**Crude OR** **(95% CI)** [n = 113]	**Adjusted OR** **(95% CI)** **[n = 78]**	**Crude β** **[n = 124]**	**Adjusted β** **[n = 85]**

Positive cockroach-induced PBMC proliferation	1.75 (0.69, 4.42)	**3.39 (1.05, 10.92)**	**0.63 (p = 0.02)**	**1.05 (p = 0.00)**
Female sex	NA	0.64 (0.22, 1.87)	NA	**-0.80 (p = 0.02)**
African American ethnicity (Reference = Dominican)	NA	01.12 (0.36, 3.46)	NA	0.30 (p = 0.37)
Age 5 ETS exposed	NA	0.83 (0.27, 2.53)	NA	-0.22 (p = 0.51)
Maternal history of asthma	NA	1.20 (0.38, 3.77)	NA	0.31 (p = 0.39)
Prenatal cockroach allergen in bed (μg/g dust)	NA	0.71 (0.46, 1.12)	NA	-0.10 (p = 0.36)

Results were based on univariate and multivariate, logistic and linear regression models. The endpoint anti-cockroach IgE is shown as odds ratio, and total IgE as regression coefficients with two-tailed p-values. Interaction terms each examined in a separate model [data not shown] demonstrated significant interactions between age 5 cockroach-induced PBMC proliferation with ethnicity (p = 0.04) and sex (p = 0.01) on age 5 anti-cockroach IgE. In stratified adjusted models, there were significant positive associations between age 5 cockroach-induced PBMC proliferation and age 5 anti-cockroach IgE amongst African Americans (β = 1.86, p = 0.00) and girls (β = 1.05, p = 0.00). Maternal asthma was defined as positive at either prenatal or 3-months postnatal time points. Cockroach prenatal allergen levels (μg/g of dust) are expressed as natural log-transformed values. OR, odds ratio. ETS, environmental tobacco smoke. NA, not applicable.

Moreover, cockroach antigen-induced PBMC proliferation was correlated weakly with total IgE production at age 5 (n = 120, Spearman's rho r = 0.225, p = 0.01). Cockroach antigen-induced PBMC proliferation was associated significantly with total IgE levels, after adjusting for potential confounders (n = 85, β = 1.05, p = 0.00) (Table 4). In contrast, mouse- and dust mite-induced PBMC proliferation did not demonstrate significant associations with antigen-specific IgE or total IgE at age 5 years (MWU Test p > 0.05). Cockroach and mouse allergen levels in bed and kitchen were not associated with antigen-specific CBMC or age 5 PBMC proliferation ratios (Kruskal-Wallis Test p > 0.05).

Positive cockroach-induced age 5 PBMC proliferation also was associated significantly with the report of physician-diagnosed asthma at age 5 (n = 131, MWU Test p = 0.03). This finding was confirmed after adjusting for the same potential confounders; age 5 children with positive cockroach-induced PBMC proliferation had three times increased odds of reported physician-diagnosed asthma at age 5 (n = 99, OR 3.08, 95% CI 1.13 to 8.37) compared to age 5 children without increased cockroach-induced PBMC proliferation (Table 5).

**Table 5:** Cockroach Antigen-Induced PBMC Proliferation and Asthma, Eczema at Age 5 Years

	Outcomes	
	
Predictors	**Asthma**^**1**^ [n = 99] OR (95% CI)	**Eczema**^**2**^ [n = 93] OR (95% CI)
Positive cockroach-induced PBMC proliferation	**3.08 (1.13, 8.37)**	2.09 (0.76, 5.75)
Female sex	0.35 (0.13, 0.92)	1.26 (0.47, 3.37)
African American ethnicity (Reference = Dominican)	2.13 (0.81, 5.63)	1.28 (0.48, 3.40)
Age 5 ETS exposed	1.36 (0.51, 3.61)	1.93 (0.69, 5.40)
Maternal history of asthma	**3.97 (1.43, 11.02)**	**3.48 (1.27, 9.56)**
Prenatal cockroach allergen in bed (μg/g dust)	0.89 (0.62, 1.27)	0.99 (0.70, 1.39)

Results were based on multivariate logistic regression models. Maternal asthma was defined as positive at either prenatal or 3-months postnatal time points. Cockroach prenatal allergen levels (μg/g of dust) are expressed as natural log-transformed values. ^1^BRQ, ^2^ISAAC Eczema Module

BRQ, Brief Respiratory Questionnaire.

ISAAC, International Study of Asthma and Allergy in Childhood.

ETS, environmental tobacco smoke

In light of research indicating that the development of eczema may be related to antigen-specific T cell immune responses [14], further cross-sectional comparisons between age 5 PBMC proliferation and prevalence of reported eczema were conducted. In univariate analysis, cockroach antigen-induced PBMC proliferation was associated with eczema at age 5 years (n = 141, OR 2.41, 95% CI 1.11, 5.22), but this association did not persist after adjusting for potential confounders (sex, ethnicity, current ETS exposure, maternal asthma, prenatal cockroach allergen levels in bed) (Table 5). Mouse- and dust mite-induced PBMC proliferation also were not associated with asthma or eczema at age 5. Moreover, concurrent cockroach-, mouse-, and dust mite-induced PBMC proliferation was not associated with cough or wheeze at age 5 (MWU Test p > 0.05 for both).

Finally, anti-cockroach, anti-mouse, but not anti-dust mite, IgE was associated significantly with concurrent asthma and wheeze at age 5 years (Asthma n = 281-283: anti-cockroach IgE, p = 0.00; anti-mouse IgE, p = 0.01; anti-dust mite IgE, p = 0.54; Wheeze n = 280-282: anti-cockroach IgE, p = 0.00; anti-mouse IgE, p = 0.00; anti-dust mite IgE, p = 0.12 by MWU Test).

## Discussion

The major objective of this study was to compare cord blood and age 5 year biomarkers such as indoor antigen-specific proliferation and cytokine production and determine whether either was associated with IgE and symptoms related to atopy and asthma in an inner city prospective cohort. We found that significant associations between antigen-specific cord blood and age 5 proliferation measures were absent. Instead, cockroach-specific proliferation assessed concurrently at age 5 years, but not in cord blood, was associated with asthma and IgE. Our focus on repeat measures and longitudinal assessment of indoor allergen responses important to inner city asthma [19,21,22] is novel.

A possible explanation for the association of age 5, but not cord blood, cockroach antigen-induced mononuclear cell proliferation with concurrent asthma and atopy may be that cord blood immune responses at birth are less mature or efficient. In support of this possibility is evidence of reduced CBMC proliferation and Th cytokine responses compared to adult responses after *ex vivo *stimulation with allergens, PHA, lipid A, and peptidoglycan [32,33]. Furthermore, there is evidence of impaired function of T regulatory cells as well as reduced expression and immature phenotype of transcription factor Foxp3, in cord blood compared to adults [32,34]. Also, T cell epitope mapping demonstrated that in response to allergen, CBMCs lack the fine specificity demonstrated by adult cells [35]. Moreover, Woodfolk et al. demonstrated evidence of differing strengths of T cell proliferative responses to *Trichophyton rubrum*, related to changes in T cell epitope recognition of the immunodominant amino-terminal that occurred over the first 2 years of life, with no change in the peptide recognition pattern after age 20 months [36]. However, it is becoming evident from our work [16] and others' [37] that the fetal adaptive immune system can be highly functional and capable of responding to antigens. Hence, an alternate explanation for the association of antigen-specific age 5 PBMC, but not CBMC, proliferation response with age 5 atopic status is that even though initial T cell priming to aeroallergens occurs across the placenta [38], clinically significant allergic sensitization to inhalant allergens occurs postnatally in early childhood [39]. Cord blood T cell responses may be specific and functional, but not necessarily committed [11].

The finding that cockroach allergen-induced proliferation at age 5 years is associated with IgE and asthma-related symptoms, as opposed to responses to other indoor allergens, is consistent with substantial research indicating that cockroach allergen is important in the pathogenesis of inner city asthma [40,41]. They also are consistent with evidence that allergen levels in home dust can be associated with allergen-induced proliferation [42]. Our additional finding that anti-cockroach IgE at age 5 years is associated both with asthma and eczema lends further support to the observed association between cockroach allergen exposure, specific allergic immune responses, and risk for asthma and atopy. Importantly, there are regional differences, as demonstrated by Matsui et al., regarding the burden of household mouse allergens on inner-city childhood asthma in Baltimore, Maryland [20]. Similarly, the association between indoor allergen-specific IgE and asthma and eczema was more apparent for dust mite in other cohorts, where dust mites thrive better, in comparison to New York City [43,44]. Further, the absence of an association between any indoor antigen-induced proliferation and eczema suggests that indoor allergen-induced T cell proliferative responses, in contrast to B cell induced responses, may not modulate the risk of developing eczema in an inner city cohort [19]. Notably, significant positive associations between age 5 cockroach-induced PBMC proliferation and age 5 anti-cockroach IgE among girls but not boys may be a result of sex specific genetic linkages as demonstrated by other groups [45,46]. Nonetheless, given the wide array and repeated immune responses to indoor antigens demonstrated by our group and others [20,40-42], public health interventions directed toward region-specific allergen reduction in the home may have health benefits to all inner-city children.

We acknowledge several limitations to this study. Atopic and respiratory symptom assessments were conducted via standardized questionnaires based upon maternal reporting. Parental report of physician diagnosis of asthma may not be standardized [26], potentially resulting in misclassification. Due to insufficient sample size, we were unable to compare antigen-specific CBMC and age 5 year cytokine responses with clinical outcomes. Furthermore, due to budgetary constraints, we were not able to measure IgE isotype class switching Th2 cytokines IL-4 and IL-13 and their association with IgE levels. In addition, due to repeated analyses, significant statistical interactions in regression models may represent type 1 error. Despite the prospective longitudinal design of the cohort, due to loss to follow-up, many data analyses were cross-sectional. Finally, host characteristics (i.e. genetics) may add to variations in the results as the development of atopy is influenced by genetic, developmental, and environmental factors [47-49].

## Conclusions

In conclusion, in contrast to cord blood, age 5 PBMC cockroach antigen-induced proliferation was associated with anti-cockroach and total IgE production and asthma in an inner-city cohort where cockroach is a prevalent allergen. If corroborated by further studies, this finding lends to potential clinical significance for use of antigen-specific proliferation assays as a biomarker for current, but not future, atopic status in early childhood.

## List of Abbreviations

BRQ: Brief Respiratory Questionnaire; CBMC: cord blood mononuclear cell; CCCEH: Columbia Center for Children's Environmental Health; CPM: counts per minute; ELISA: enzyme-linked immunosorbent assay; ETS: environmental tobacco smoke; FAST: Fluorescence Allergosorbent Test; IL: interleukin; IRMA: immunoradiometric assay; ISAAC: International Study of Asthma and Allergy in Childhood; MUP: mouse urinary protein; MWU: Mann-Whitney U; PBMC: peripheral blood mononuclear cell; PHA: phytohemagglutinin; SI: stimulation index; SPT: skin prick test; TH: T helper

## Declaration of Competing interests

The authors declare that they have no competing interests.

## Authors' contributions

CC conceived of the study with RLM, and participated in its design and coordination, its statistical analysis and helped to draft the manuscript. KGK participated in the conduction and coordination of the study and helped to draft the manuscript. AJ participated in the conduction and coordination of the study. EAK participated in its design and its statistical analysis. GLC participated in the design of the study. FP conceived of the study with RLM, and participated in its design and coordination. RLM conceived of the study, and participated in its design and coordination and statistical analysis and helped to draft the manuscript. All authors read and approved the final manuscript.

## Acnowledgements

Funding was provided by: The National Institute of Environmental Health Science (P01ES09600, R01ES008977, R01ES13163), and U.S. Environmental Protection Agency (R827027).
